# Does online learning work better than offline learning in undergraduate medical education? A systematic review and meta-analysis

**DOI:** 10.1080/10872981.2019.1666538

**Published:** 2019-09-17

**Authors:** Leisi Pei, Hongbin Wu

**Affiliations:** a Institute of Basic Education Science, Beijing Academy of Educational Sciences, Beijing, China; b Institute of Medical Education/National center for Health Professions Education Development, Peking University, Beijing, China

**Keywords:** Online learning, offline learning, systematic review, meta-analysis, undergraduate medical education (UME)

## Abstract

With the increasing use of technology in education, online learning has become a common teaching method. How effective online learning is for undergraduate medical education remains unknown. This article’s aim is to evaluate whether online learning when compared to offline learning can improve learning outcomes of undergraduate medical students. Five databases and four key journals of medical education were searched using 10 terms and their Boolean combinations during 2000–2017. The extracted articles on undergraduates’ knowledge and skill outcomes were synthesized using a random effects model for the meta-analysis.16 out of 3,700 published articles were identified. The meta-analyses affirmed a statistically significant difference between online and offline learning for knowledge and skill outcomes based on post-test scores (SMD = 0.81; 95% CI: 0.43, 1.20; p < 0.0001; n = 15). The only comparison result based on retention test scores was also statistically significant (SMD = 4.64; 95% CI: 3.19, 6.09; p < 0.00001). The meta-analyses discovered no significant difference when using pre- and post-test score gains (SMD = 3.03; 95% CI: −0.13, 4.13; p = 0.07; n = 3). There is no evidence that offline learning works better. And compared to offline learning, online learning has advantages to enhance undergraduates’ knowledge and skills, therefore, can be considered as a potential method in undergraduate medical teaching.

## Introduction

Today, digital information is everywhere and available to almost everyone. In this era of information technology, medical education is now confronted with novel challenges. On the one hand, the online healthcare information boom is continually challenging medical students to rapidly update and expand their existing body of knowledge. On the other hand, the informatic competency requirements of healthcare technology, such as utilizing electronic healthcare records, learning systems and aided-diagnosis systems, also present a new challenge for medical students to master [1], even for the so-called digital native learners [2].

To prepare qualified doctors for today’s environment in which the internet provides ubiquitous digital information, the teaching methods used for educating and training medical school students should be reconsidered. Offline learning, or traditional classroom teaching, represents teaching in the pre-internet era. Although some forms of information technology have already been utilized to assist instruction, traditional teaching methods required that teaching and learning should take place at the same time and place. Online learning, also called internet-based learning or web-based learning, does not have the time and space limitations, and therefore, makes teaching and learning separable via internet-based information delivery systems. Both online and offline teaching have been widely used in higher education. The use of online learning has vastly increased since 2012, as evidenced by the thriving of massive open online courses (MOOCs) [3]. However, evaluating the effectiveness of online and offline teaching remains difficult. Evaluations have failed to reach consistent conclusions [4,5], resulting in complex decisions when selecting a teaching method for medical education.

The effectiveness of online learning is influenced by many factors. Some factors create barriers for online learning, such as administrative issues, social interaction, academic skills, technical skills, learner motivation, time and support for studies, technical problems, cost and access to the internet [6]. Other factors could result in low-quality online learning, for example an ineffective design and arrangement of multimedia materials [7]. The effective analysis of online and offline teaching in medical education, therefore, should depend on a comprehensive consideration of how they are used across groups. It should all be assessed including the learning goals, design properties of the learning materials, evaluation of learning outcomes, etc.

The first comprehensive systematic review and meta-analysis of online versus offline learning dates back to 2008. Cook et al. [4] selected 76 articles that compared internet and non-internet based interventions and 130 articles containing no-intervention control for health professional learners. Through a meta-analysis, this study concluded that internet-based interventions were associated with positive effects compared to no interventions, but the effects and statistical heterogeneities were generally small compared to offline teaching. Richmond et al. [8] then updated the evidence in this rapidly developing field by subdividing different formats of offline learning and comparing these formats to online learning. They focused their review, as well, on licensed healthcare professionals. However, this more recent evidence still suggests that online learning might be as effective as offline one for training licensed healthcare professionals, but the total effects of the online learning were low and showed no significant difference when compared to offline teaching.

Accordingly, the current meta-analysis was designed to contribute additional evidence from a new perspective in the comparison of the intervention effects of online learning versus offline learning. In contrast to previously published reviews, our analysis narrowed the target participants to undergraduate medical students and excluded postgraduates and professionals like nurses, pharmacists, veterinarians, etc. The reason why we concentrate on this specific group is that different from postgraduates’ self-motivated and clinic practice-orientated learning, undergraduate medical students are mainly driven by common core curricula and examinations stipulated by the universities’ teaching committee, which reversely, brings a sharp gap when evaluating teaching methods on these two groups of students, respectively, [9]. Moreover, our study design concentrated on knowledge and skill outcomes but distinguished among different statistical methods used when generating comparison results. By testing whether online learning worked better than offline one for medical undergraduate education, this review also intended to preliminarily explore the potential factors across these two teaching methods that might cause differences in effectiveness. Identifying such differences could have implications for further research and improvements in educational practices.

## Methods

### Study design

The preferred reporting items for systematic reviews and meta-analyses (PRISMA) guidelines and recommendations from the Cochrane Handbook were followed [10,11]. There were no requirements for an ethical review of this paper since no human participants were involved.

The objective of this systematic review was to assess how online learning compared to offline learning for teaching the medical education knowledge and skills.

### Literature sources and searches

The Web of Science, Medline, Embase, PubMed and Scopus were searched for the following terms in the title and abstract: (online learn* OR web-based OR internet OR m-learning OR mobile OR distance) AND (medical edu*) AND (student* OR undergraduat* OR universit*). Four key journals of medical education, Medical Teacher, Academic Medicine, Medical Education and BMC Medical Education, were manually searched for relevant articles.

We used a search start date of January 2000 and an end date of December 2017. Because digital technologies have undergone dramatic changes since the internet first appeared in 1991 [12] and internet-based hard facilities and soft applications in education have been widely accepted by schools and students starting in the 21^st^ century [13], we therefore restricted the start date to after the year 2000. The search was reconducted on May 1^st^, 2019.

### Inclusion criteria

The included studies should meet the following criteria in adherence to the participant, intervention, comparison and outcome (PICO) search in the field of evidence-based medicine:

- Participants: medical undergraduate students.
- Interventions: online learning, including e-learning, m-learning, MOOCs and distance learning by video.
- Comparisons: offline learning, especially referring to face-to-face teaching in a classroom, seminars, watching video lectures together in the classroom and reading text-based documents or books only.
- Outcomes: knowledge and skill outcomes measured by objective assessment instruments. The mean score and standard deviations of post-test, pre- and post-test gains, or retention tests for experimental and control groups were available.

### Data screening and extraction

The titles of the retrieved articles were first screened by a reviewer (P) based on the inclusion criteria. Duplicates and studies that were superficially unassociated with the comparison of online learning and offline learning were excluded. Then, the abstracts of the remaining articles were independently screened by two reviewers (P and W) based on the criteria. Any articles that seemed to be dubious based on the abstract screening were further examined by reading the full text. In the full-text screening phase, the two reviewers again worked independently to review every article against the criteria. Any conflicts between the two reviewers were resolved by consensus.

### Quality assessment

The quality of methodology used in each article was evaluated based on the Medical Education Research Study Quality Instrument (MERSQI) [14].

The risk of bias was assessed according to the Cochrane collaboration risk of bias assessment tool [11], which contains random sequence generation (selection bias), allocation concealment (selection bias), blinding of participant and personnel (performance bias), blinding of outcome assessment (detection bias) and incomplete outcome data (attrition bias). For each of these items, the judgment of ‘low risk of bias,’ ‘unclear risk of bias’ and ‘high risk of bias’ was given with necessary supporting statements for each article.

### Data synthesis

We classified the identified articles based on the statistical method of outcome, including analysis of variance (ANOVA) on post-test scores, pre- and post-test score gains and delayed retention scores. When an article contained more than one statistical method of outcome, it was clustered repeatedly into a different genre of the meta-analysis. For those articles that included multiple arms but used the same statistical method, we first considered each of the comparisons, respectively, in the meta-analysis. Then, we only included one comparison result under one genre of meta-analysis in each article, because including multiple comparison results from the same article obviously does not meet the criteria of statistical independence [15].

The standard mean difference (SMD) with 95% confidence intervals (CIs) was applied for the overall effect of group comparisons in the meta-analysis. The statistical heterogeneity was calculated using the *I^2^* statistic [16]. For a high heterogeneity value (*I^2^*> 50%) [17], the recommended random-effects model was used in the meta-analysis for the pool weighted effect sizes [18]. The effect sizes were interpreted as 0.2 for a small effect, 0.5 for a moderate effect and 0.8 or greater for a large effect [8]. We used Review Manager (*RevMan 5.3*) [19] to carry out the meta-analyses in this review.

## Results

### Search results

**Figure 1.**
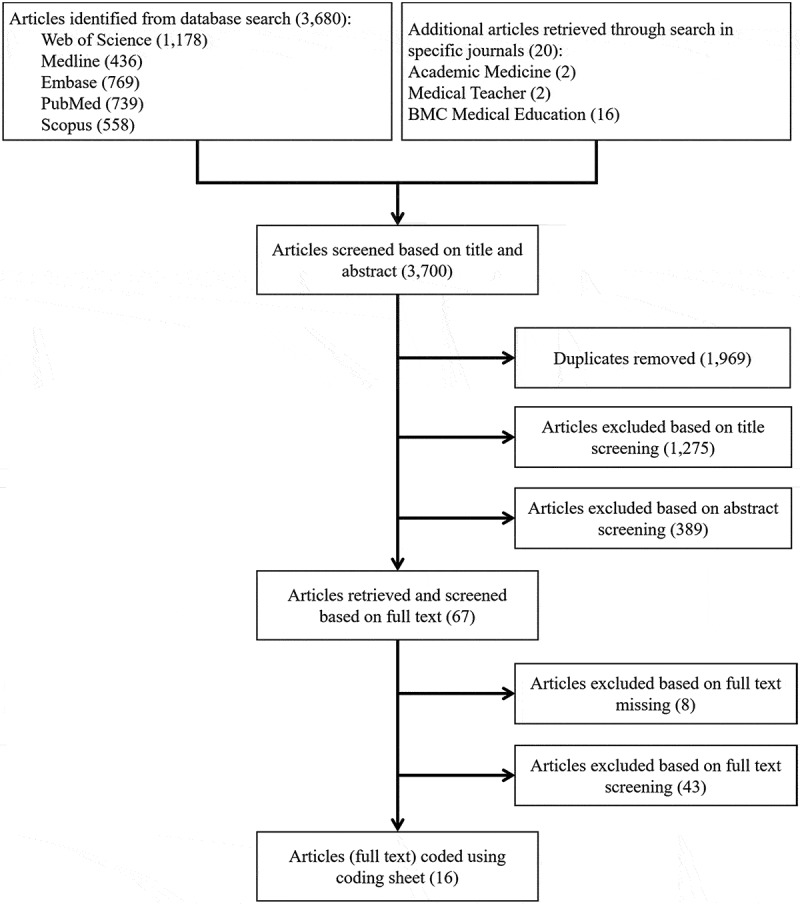
Study inclusion flowchart.

The flowchart of article inclusion is shown in Figure 1. A total of 3,680 articles were searched in five databases, and additional 20 articles were retrieved by searching four specific journals. Among them, 1,969 duplicates were removed manually, 1,275 articles were excluded based on title screening, and 389 articles were excluded based on abstract screening against the inclusion criteria. Sixty-seven full articles were then screened. However, 8 of them could not be accessed in the full text, and 43 articles were excluded against the inclusion criteria. Finally, 16 articles were remained for this systematic review.

### Methodological quality

The mean (SD, range) of the total score for included articles was 13.5 (1.1, 11–15) of 18 (Table 1). All included articles used appropriate data analysis methods. Only two articles sampled from more than one institution [20,21]. Some of the included articles were rated lower than others, due to a lower score in the ‘validity of educational instrument’ domain (mean (SD) score of 1.5 (0.8) out of 3).

**Table 1.** Methodological quality of included studies.

		Study	Score		Mean (SD)	
Domain	MERSQI Item	NO. (%)	Item	Maximum Domain	Item	Domain
Study design	1. Study design			3	2.8 (0.6)	28 (0.6)
	Single group cross-sectional or single group post-test only	1 (6%)	1			
	Single group pre-test and post-test		1.5			
	Non-randomized, two group	2 (13%)	2			
	Randomized control trail	13 (81%)	3			
Sampling	2. No. of institutions studied			3	0.6 (0.2)	2.0 (0.3)
	1	14 (88%)	0.5			
	2	2 (13%)	1			
	>2					
	3. Response rate, %				1.4 (0.2)	
	N/A					
	<50 or not reported		0.5			
	50-74	2 (13%)	1			
	≥75	14 (88%)	1.5			
Type of data	4. Type of data			3	3.0 (0.0)	3.0 (0.0)
	Assessment by study participant		1			
	Objective measurement	16 (100%)	3			
Validity of evaluation instrument	5. Internal structure			3	0.8 (0.4)	1.3 (0.7)
	N/A					
	Not reported	4 (25%)	0			
	Reported	12 (75%)	1			
	6. Content				0.2 (0.4)	
	N/A					
	Not reported	13 (81%)	0			
	Reported	3 (19%)	1			
	7. Relationship to other variables				0.3 (0.5)	
	N/A					
	Not reported	11 (69%)	0			
	Reported	5 (31%)	1			
Data analysis	8. Appropriateness of analysis			3	1.0 (0.0)	3.0 (0.0)
	Data analysis inappropriate for study design or type of data		0			
	Data analysis appropriate for study design or type of data	16 (100%)	1			
	9. Complexity of analysis				2.0 (0.0)	
	Descriptive analysis only		1			
	Beyond descriptive analysis	16 (100%)	2			
Outcomes	10. Outcomes			3	1.5 (0.0)	1.5 (0.0)
	Satisfaction, attitudes, perceptions, opinions, general facts		1			
	Knowledge, skills	16 (100%)	1.5			
	Behaviors		2			
	Patient/healthcare outcome		3			
Total score				18	13.5 (1.1)	

### Risk of bias

**Figure 2.**
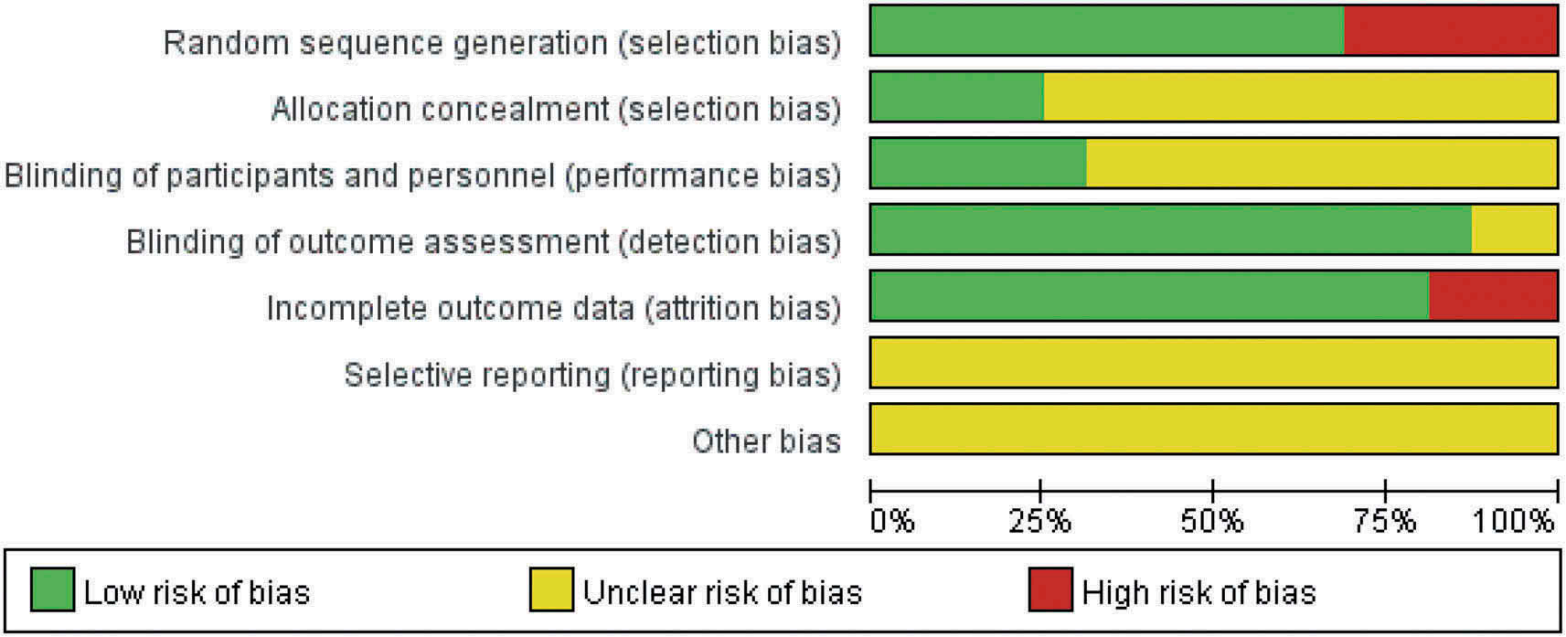
Summary of the risk of bias.

The overall risk of bias for all the included articles is shown in Figure 2, and Table 2 shows a detailed judgment of the risk of bias for each article. Six domains of bias were evaluated, and no article reported information about the ‘reporting bias’. Very few articles described the randomization process in detail, which possibly could be that authors considered such a description as unnecessary, and opted instead to just use the word ‘randomized.’ The true blinding of participants was nearly impossible to achieve as well because most of researchers had to describe the study for participant recruitment. However, some studies achieved participant blinding by recruiting students in different academic years as experimental and control groups, using a crossover study design or providing randomized materials.

**Table 2.** Risk of bias.

	Selection bias		Performance bias	Detection bias	Attrition bias	Reporting bias	
Reference	Random sequence generation	Allocation concealment	Blinding of participants and personnel	Blinding of outcome assessment	Incomplete outcome data	Selective reporting	Other bias
Solomon et al. (2004)	Low – ‘ … were randomized into to … ’	Unclear – Insufficient information	Unclear – Insufficient information	Unclear – Insufficient information	Low – All participants assessed	Unclear – Insufficient information	Unclear – Insufficient information
Phadtare et al. (2009)	Low – ‘Random numbers were generated with … based on program of origin’	Low – ‘Group assignments were placed in sealed envelopes and revealed after participants had signed informed consent’	Low – ‘To ensure blinding, assignments were disclosed to analysts only after the results had been delivered’	Low – ‘To ensure unbiased findings, statistical analysis was blinded, with analysts being unaware of which group participants were assigned to until the study analysis was complete’	Low – All participants assessed	Unclear – Insufficient information	Unclear – Insufficient information
Raupach et al. (2009)	Low – ‘Students who had signed up together were randomized as a group to either the control or the intervention setting’	Unclear – Insufficient information	Unclear – Insufficient information	Low – ‘On the last day, all students took a summative examination made up of 68 multiple choice questions mainly assessing factual knowledge’	Low – ‘5 out of 148 participants dropped out’	Unclear – Insufficient information	Unclear – Insufficient information
Bhatti et al. (2011)	Low – ‘The students were randomly assigned to either group A or group B using QUICKCALCS online software’	Unclear – Insufficient information	Low – ‘Students were made aware that they had taken part in a study to compare educational methods, but they were not told until after the information had been delivered’	Low – ‘The papers were marked by an individual blinded to the teaching method given, using a pre-agreed marking schedule’	High – ‘121 out of 146 participants completed the study’	Unclear – Insufficient information	Unclear – Insufficient information
Heiman et al. (2012)	Low – ‘Upon matriculation, students were assigned randomly to one of four colleges’	Unclear – Insufficient information	Low – ‘All second-year students were randomly assigned a case from the bank of six assessment cases’	Low – ‘Raters were paid per case completed. They were blinded to the training status of students but not to the timing of the evaluation’	Low – all participants assessed	Unclear – Insufficient information	Unclear – Insufficient information
Serena et al. (2012)	High – ‘Participants are enrolled in different semester year’	Unclear – Insufficient information	Low – ‘Participants in the two groups were in different academic years’	Low – ‘An independent scorer applied the rubric to all pre- and post-tests’	Low – All participants assessed	Unclear – Insufficient information	Unclear – Insufficient information
Subramanian et al. (2012)	Low – ‘Medical students were consented and randomly assigned to two groups’	Unclear – Insufficient information	Unclear – Insufficient information	Low – ‘A bank of multiple-choice questions was created. The questions were randomly selected for a preintervention test and postintervention test’	Low – All participants assessed	Unclear – Insufficient information	Unclear – Insufficient information
Yeung et al. (2012)	Low – ‘Randomization and allocation concealment were achieved through an automatic randomization process’	Low – ‘Randomization and allocation concealment were achieved through an automatic randomization process’	Low – ‘Access to each module was restricted to the individuals randomized to each respective study group’	Low – ‘The primary outcome measure was a multiple-choice quiz’	Low – All participants assessed	Unclear – Insufficient information	Unclear – Insufficient information
Jordan et al. (2013)	High – This is a single group cross-sessional study	Unclear – Insufficient information	Unclear – Insufficient information	Low – “A multiple choice post-test was used to assess their knowledge gain”	Low – ‘4 out of 48 participants dropped out’	Unclear – Insufficient information	Unclear – Insufficient information
Sendra et al. (2013)	High – “The project was accepted by 89 students out of 191 (46.6%), who integrated the group P, attending only virtual lectures. The remaining 102 students (53.4%) did not participate in the project, being the control group NP. “	Unclear – Insufficient information	Unclear – Insufficient information	Low – ‘The final oral examination and an anonymous evaluation on image interpretation, where the name of the students remained unknown’	High – ‘74 out of 89 in group P and 56 out of 102 in group NP’	Unclear – Insufficient information	Unclear – Insufficient information
Porter et al. (2014)	Low – ‘All students who enrolled in the course through the preregistration process were randomly assigned to either the classroom or online section using block randomization’	Unclear – Insufficient information	Unclear – Insufficient information	Low – ‘The lecturing faculty member was blinded to the participation status of the students.’	High – ‘140 students participated in the study, which is a participation rate of 83.3%.’	Unclear – Insufficient information	Unclear – Insufficient information
Assadi et al. (2015)	High – ‘Divided into two groups by odd and even month’	Unclear – Insufficient information	Unclear – Insufficient information	Low – ‘Evaluation of participants was assessed by an EM attending (M.M.) who was blinded to the training methods.’	Low – ‘9 out of 90 interns were not available.’	Unclear – Insufficient information	Unclear – Insufficient information
Pusponegoro et al. (2015)	Low – ‘Subjects were randomized into two groups using a computer-generated random number table’	Unclear – Insufficient information	Unclear – Insufficient information	Low – ‘Complete a 20-item multiple-choice test’	Low – ‘4 out of 75 participants dropped out’	Unclear – Insufficient information	Unclear – Insufficient information
Arne et al. (2016)	Low – ‘The allocation to the various branches of the study was carried out by randomization’	Low – ‘All students were anonymously assigned in advance, with a number (“token”) that was used for identification purposes throughout the study’	Unclear – Insufficient information	Low – ‘These tests were based on a 24-item multiple-choice questionnaire. Each question included five possible answers, of which only one was correct’	Low – ‘21 out of 244 participants dropped out’	Unclear – Insufficient information	Unclear – Insufficient information
Farahmand et al. (2016)	High – ‘This was a blinded quasi-experimental study’	Low – ‘To conceal the allocation, the first group, who started their emergency medicine rotation in September to October 2013, entered the control group and the nature of the future intervention was not revealed to them. We did not inform them about the existence of the educational DVD’	Unclear – Insufficient information	Low – ‘Both groups and raters who scored the students during the OSCE were blinded to the content of the educational package and the intervention of each group’	Low – All participants were assessed	Unclear – Insufficient information	Unclear – Insufficient information
Shenoy et al. (2016)	Low – ‘Students were randomly divided into two groups’	Unclear – Insufficient information	Unclear – Insufficient information	Unclear – Insufficient information	Low – All participants were assessed	Unclear – Insufficient information	Unclear – Insufficient information

### Synthesis of included articles

The total number of recruited participants in all the comparison results without duplications was 1,789 and the mean and range were 112 and 29–294, respectively (Table 3). The included comparisons were conducted in nine countries (USA, UK, Spain, Brazil, Germany, China, Iran, Indonesia and India).

**Table 3.** Details of included studies.

Reference	Method	Population	Intervention and comparison	Outcome	Finding
Solomon et al. (2004)	Randomized controlled trial, USA	29 third-year students who had completed an internal medicine rotation	Attended a lecture series on campus or viewed digital versions of the same lectures at community-based teaching sites.	The same short examination that included 4–5 questions based on lectures, live group answered in written form but digital group in digital form.	No differences in performance as measured by means or average rank.
Phadtare et al. (2009)	Randomized controlled trial, USA and Brazil	48 second- and third-year medical students	Received standard writing guidance in a classroom setting or an online writing workshop.	Manuscript quality was evaluated according to well-defined parameters using the Six-Subgroup Quality Scale, and self-reported satisfaction scores were evaluated using a Likert scale.	Online scientific writing instruction was better than standard face-to-face instruction in terms of writing quality and student satisfaction.
Raupach et al. (2009)	Randomized controlled trial, Germany	148 fourth-year medical students enrolled in the 6-week course	Diagnosed a patient complaining of dyspnea using either a virtual collaborative online module or a traditional problem-based learning (PBL) session.	Clinical reasoning skills were assessed with a key feature examination at the end of the course.	No significant difference between the mean scores of both study groups. The evaluation data favored traditional PBL sessions over virtual collaborative learning.
Bhatti et al. (2011)	Randomized controlled trial, UK	148 third-year medical students starting their first clinical rotation	Group A was given a lecture and group B was asked to use a website containing text and pictures that was augmented by a podcast.	Pre-intervention questionnaire for baseline knowledge, the same questionnaire for post-intervention test and satisfaction was acquired with a feedback form.	No differences in knowledge at baseline, significant post-test increase in knowledge for group B (web-based). Both groups were equally satisfied with the educational method.
Heiman et al. (2012)	Randomized controlled trial, USA	132 second-year medical students	Received a web-based, interactive curriculum or control.	Evaluated students’ performance of presentations at three time points.	Significant difference in the presentation performance between the groups, with the online group significantly improved.
Serena et al. (2012)	Non-randomized two groups, USA	56 students in 2004–05 academic year for control group, and 111 students in 2005–06 academic year for intervention group	Received a 1-hour live lecture on delirium or completed the online delirium curriculum.	A short-answer test with two cases was used for the pre- and post-test.	No significant difference in the test score improvement between the two groups.
Subramanian et al. (2012)	Randomized controlled trial, USA	30 third-year medical students	Listened to two 30-min PowerPoint-based lectures about torsades de pointes (TdP) and pulseless electrical activity (PEA), or reviewed 2 cases in a web-based medical learning modality on TdP and PEA over 1 hour.	A 20-question, multiple-choice pre-intervention test assessed baseline knowledge, a 40-question post-intervention test assessed understanding of the recognition and management of TdP and PEA and a 22-of-40-question long-term retention test assessed retention 22 d later.	The web-based learning group demonstrated a significant improvement in retention compared to the group that received the traditional didactic lecture format.
Yeung et al. (2012)	Randomized controlled trial, UK	78 undergraduate anatomy students	Accessed a computer-assisted learning (CAL) module or traditional text-/image-based learning supplements.	A multiple-choice knowledge quiz was used for skill evaluation.	No significant difference was identified. The CAL modules might have helped pique student interest and motivation.
Jordan et al. (2013)	Observational quasi-experimental, USA	44 of 48 fourth-year medical students enrolled in the 2011–12 course	Received computer-based modules via the internet or attended traditional lectures.	A multiple-choice test for pre- and post-test and a retention test were used to assess knowledge gain, and a five-point Likert scale questionnaire was used to assess attitude.	The knowledge gain in the group that was instructed with the didactic method was significantly higher than the computer-based group. There was no significant difference in the retention test scores.
Sendra et al. (2013)	Non-randomized two groups, Spain	89 of 191 third year students in the 2005–06 academic year elected to be in the participant group, and the remaining students were the non-participant group	Received 22 virtual lectures or conventional lectures.	A final oral exam and a 60-question evaluation on image interpretation were used.	Final exam qualifications were significantly higher for the virtual lecture group.
Porter et al. (2014)	Randomized controlled trial, USA	198 second- and third-year students	Assigned to either the classroom or online section.	28-question survey instruments about demographic information and course delivery were used for pre- and post-intervention assessment.	No significant difference was found for any of the grades in the course.
Assadi et al. (2015)	Non-randomized two groups, Hong Kong, China	81 undergraduate medical interns	Received a DVD containing a 20-min training video or took part in a 4-hr training class.	A pre- and post-test based on the 2010 American Heart Association resuscitation guidelines.	The video group achieved slightly better scores compared to the traditional group.
Pusponegoro et al. (2015)	Randomized controlled trial, Indonesia	71 fifth-year medical students	Received online video modules, discussions and assessments or received a 1-day live training using the same module.	Both attended pre- and post-tests and completed the User Satisfaction Questionnaire (USQ). The web-based group also completed the System Usability Scale (SUS).	Pre- and post-test scores did not differ significantly between the two groups. Both training methods were acceptable based on the USQ scores. The web-based training had good usability based on the SUS scores.
Arne et al. (2016)	Randomized controlled trial, mixed methods study, Germany	223 out of 244 medical students in the third academic year	Received one of four learning forms: self-instructed learning (e-learning and curriculum-based self-study) and instructed learning (lectures and seminars).	A multiple-choice questionnaire was used for pre- and post-test, and a self-assessment was used for satisfaction and learning style.	The students in the modern study curricula learned better through self-instruction methods. There were good levels of student acceptance and higher scores in personal self-assessment of knowledge.
Farahmand et al. (2016)	Non-randomized two groups, Iran	120 senior medical students	One group attended a workshop with a 50-min lecture and a case simulation scenario followed by a hands-on session. The other group was given a DVD with a similar 50-min lecture and a case simulation scenario and also attended a hands-on session.	A 25-question multiple-choice test evaluated the basic knowledge before the intervention. An objective structured clinical examination evaluated the performance.	The performance in the distance learning group was significantly better.
Shenoy et al. (2016)	Randomized controlled trial, crossover study Kottayam, India	147 first-year MBBS students	First round: the first group attended a conventional lecture, and the second group received e-learning. Second round: the first group received e-learning, and the second group attended a conventional lecture.	Students’ perception of e-learning was assessed by a validated questionnaire and performance by a post-test.	Compared to the conventional teaching method, e-learning was significantly different in in terms of post-test marks and was liked by 72.8% of the students.

**Figure 3.**
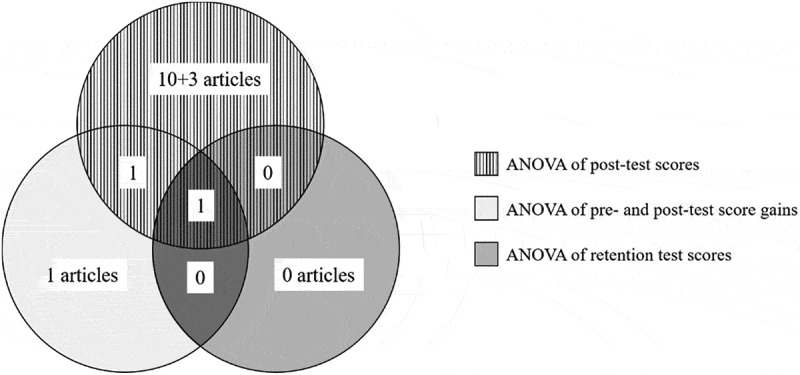
Venn diagram of the 16 identified articles, clustered by the statistical methods used.

Sixteen identified articles were clustered by the statistical methods used (Figure 3), specifically by the number of outcome comparisons. Among them, 13 articles reported only one comparison arm based on one or more statistical method based on ANOVA: 10 articles compared post-test scores [20–29]; 1 article compared pre- and post-test score gains [30]; 1 article compared both post-test scores and pre- and post-test score gains on the same sample [31]; and 1 article compared all post-test scores, pre- and post-test score gains and retention test scores on the same sample [32]. All of comparison results above were included in meta-analysis but assessed under different genres. The remaining 3 articles contained more than one comparison arm but were all based on ANOVAs of post-test scores: 2 articles reported 2 comparisons using 2 different measure instruments [33,34]; and 1 article reported 2 comparison results for 2 different learning goals on the same sample [35]. To ensure statistical independence, we only extracted one comparison result from each article for the meta-analysis. However, it is worth noting that Jordan et al. [30] reported both post-test scores and pre- and post-test score gains. We only used the latter one because the baselines of the two groups were significantly different. Overall, 15 comparison results were extracted for post-test scores, 3 for pre-and post-test score gains and 1 for retention test scores.

Although all of included articles assessed knowledge or skill outcomes in medical education, there was no overlap among them. It is also worth noting that 2 articles assessed knowledge or skill outcomes that are not that specific to medical education: scientific writing [20] and oral case presentation [21].

The intervention durations also varied among the included articles, ranging from about 20 min to an academic semester (around 18 weeks). And one article did not report the duration.

The formats of online learning used were also various in the studies. The simplest format consisted of a CD-/DVD-based video lecture that was recorded from a live class and then uploaded to the internet, and the most advanced format was a platform that allowed students to receive static learning resources and facilitated interaction with teachers, classmates and courseware for responsive feedback.

#### Meta-analysis based on post-test scores

**Figure 4.**
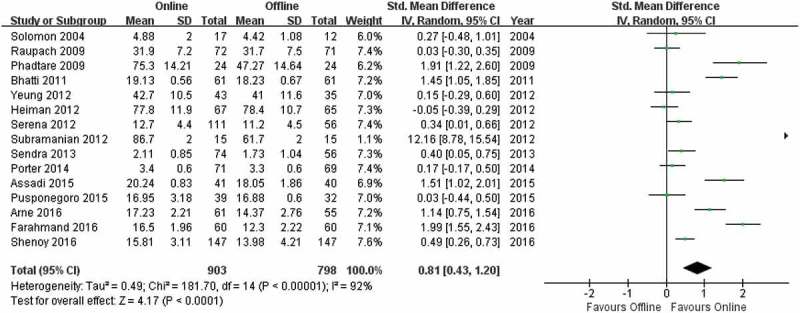
Meta-analysis of post-test performance.

Figure 4 shows the two groups were significantly different (Z = 4.17; p < 0.0001), with the online learning group having higher post-test scores (SMD = 0.81; 95% CI: 0.43, 1.20).

**Figure 5.**
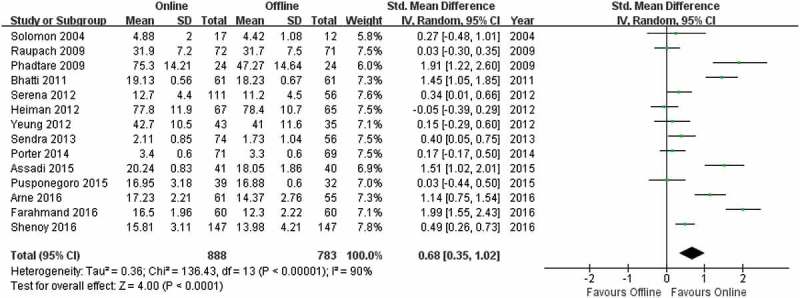
Meta-analysis of post-test performance without the article of Subramanian et al.

Subramanian et al. [32] reported a larger SMD than the other articles, and this value could contribute heavily to the summary effect in the meta-analysis. To avoid a potential determinative influence from this single article, we conducted a separate meta-analysis on the post-test scores without this study (Figure 5). There was still a significant difference (Z = 4.00, p < 0.0001) between the online learning and offline learning groups, favoring online learning (SMD = 0.68; 95% CI: 0.35, 1.02).

#### Meta-analysis based on pre- and post-test score gains

**Figure 6.**
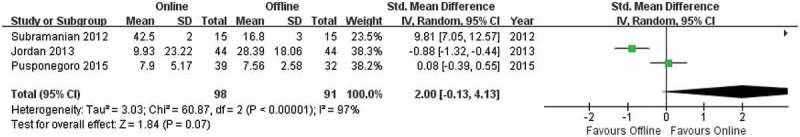
Meta-analysis of pre- and posttest score gains.

Although there was no significant difference between the groups (Z = 1.84; p = 0.07), but the gains in online learning groups trended higher (SMD = 2.00; 95% CI: −0.13, 4.13, Figure 6).

#### Meta-analysis based on retention test scores

**Figure 7.**
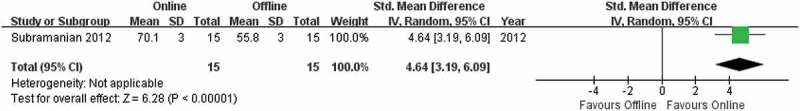
Meta-analysis of retention test scores.

The article of Subramanian et al. [32] was the only study to compare retention test scores. Online learning (70.1 ± 3%) was more effective than offline learning (55.8 ± 3%) with a statistically significant difference (SMD = 4.64; 95% CI: 3.19, 6.09; p < 0.00001, Figure 7).

## Discussion

In this review, reliable evidences from 2000 to 2017 were scrutinized and synthesized to answer the question: does online learning work better than offline learning for undergraduate medical students? We screened 1,731 unduplicated articles and eventually identified 16 articles that meeting the inclusion criteria. By comparing post-test scores, pre- and post-test score gains and retention test scores, we identified 7 articles that reported no significant difference between the two teaching methods and 9 articles that reported significant improvement in the online learning groups. Whether we included the article of Subramanian et al. [32] or not, the changes in post-test scores indicated that online learning for medical education might be more effective than offline learning when assessed based on the outcomes of knowledge and skills. The examination of the effects on pre- and post-test score gains showed little difference between these two methods. The article of Subramanian et al. [32] was the only study to use a delayed retention test, which showed online learning was better than offline learning. Overall, it suggested that online learning is at least as effective as offline learning, however we still need more research evidences to draw any firm conclusion on the comparison of online versus offline learning, since experimental designs of the included articles varied in terms of participants, learning goals, intervention durations, and forms of online learning, etc.

Although the overall finding indicated that online learning worked as well as offline learning, it didn’t imply that online learning is an effective teaching method for every student in every learning context. We note the effects of online learning reported in the article of Subramanian et al. [32] stood out against the results in the other 15 articles. Through a comprehensive evaluation, we determined that the format of online learning used by Subramanian’s team, StepStone Interactive Medical Software, might have played a key role in that study, since the rich feedback and guidance, matched task difficulties to students’ developmental level [36] and case-based teaching strategies designed for online learning might improve the outcomes of online learning. For online learning that consisted mainly of static, non-interactive learning resources that largely resembled offline learning, usually no significant difference was found when compared to offline learning. In addition, the knowledge and skills taught in the included studies actually only covered a small part of the learning goals in medical education. It is highly possible that online learning might not work better than offline learning for the topics that remain to be studied. Moreover, the objective assessment instruments used in the articles might not be able to evaluate the advanced capacities acquired by undergraduate medical students. Given that the objective assessments filled with multiple choice questions were more appropriate for the assessment of low-level learning goals, online learning, therefore, might only be as effective as offline learning when the learning goals are simple. Similarly, type of curriculum, usually associating largely with learning goals, might also affect the effectiveness of online and offline learning; however, it is known that undergraduate medical courses emphasized mainly on basic knowledge and skills, we still cannot speculate whether online works better than offline learning across various curriculum types before new evidence emerges. Besides above, the effectiveness could also be influenced by characteristics of students themselves, such as gender, learning style [37], attitude [38], satisfaction [39] and level of engagement [40].

The rapid growth of online learning in higher education has also benefited from the potential cost savings for limitless students [41]. The undergraduates who participated in the included studies were passively arranged into an experimental or control group, and they did not have to figure out how to pay for the teaching they received, which is not realistic. A recent study, conducted in a large for-profit university with an undergraduate enrollment of more than 100,000, estimated the effects of online learning and face-to-face instruction on students’ achievement and progress. As a result, students got lower grades for both the course taken online and the courses that followed [42]. Therefore, the choice of teaching method should also be made after comprehensive thought of human economic behaviors in the real world.

To some extent, online learning might not compete with some aspects of offline learning, like interactive knowledge building between teacher and students. Such limitations could create opportunities for students to obtain self-learning abilities through information technology, such as information literacy and metacognition controlling [43].

The effectiveness of online learning varied, which is as or more effective than offline learning for some target knowledge and skills and also the students. To avoid the potential limitations of online learning in undergraduate medical education, it might be worthwhile to combine the advantages of online and offline teaching methods, called blended learning [44]. Despite the uncertainties of online learning, it should be allowed in undergraduate medical education, but to maximize the benefits, a combination of online and offline learning might be the most effective.

### Limitations

There are still some limitations of this study. First, the small number of included studies. Although we actually used a relatively broad search strategy, but when narrowed down based on the inclusion criteria, only 16 articles were eventually identified and the total number of participants was 1,789, including 947 in online learning groups and 842 in offline learning groups. It should also be emphasized that the meta-analyses did not differentiate knowledge outcomes from skill outcomes [45] but regarded these two categories of outcomes as equal. What was discriminated were the statistical methods. Second, the different statistical heterogeneities of the meta-analyses with and without the article of Subramanian et al. [32] complicated conclusions about the effectiveness of online versus offline learning.

### Further research

Despite some outstanding questions, the findings of this review offer supporting evidence on the effectiveness of online learning in undergraduate medical education. Further research is needed to clarify the effects of online learning and the conditions under which it can be effectively used. Whether online learning works as a direct or mediated factor in improving achievement needs to be assessed, as do what design and delivery strategies for online learning works in practice. How the advantages of online learning can be used to amplify other teaching methods for undergraduate medical students also needs to be studied. The design of the assessment instruments and curriculum types used for online learning requires further study. It is possible that students do acquire knowledge and skills through online learning that cannot obtain through offline learning, and this knowledge could compensate for the loss of knowledge and skills identified by questionnaires for offline learning.

## Conclusion

Although not all of the included research studies reported that using online learning methods in medical education was more effective than offline learning, none of the included studies concluded that online learning was less effective than offline methods, regardless of the statistical method used. We need to recognize that online learning has its own advantages for enhancing students’ learning and should be considered a potential teaching method in medical education. To guarantee the effectiveness of online learning, the design principles of digital learning materials, learning goals and students’ preferences and characteristics should be rigorously evaluated.
